# Selective Fetal Growth Restriction Leading to Cerebral Injury in Monochorionic Twins: A Case Report

**DOI:** 10.7759/cureus.75387

**Published:** 2024-12-09

**Authors:** Patricia Campos, Ana R Matos, Ana Ferraz, Raquel Henriques

**Affiliations:** 1 Neonatology Department, Daniel de Matos Maternity, Coimbra Local Health Unit, Coimbra, PRT

**Keywords:** cerebral injury, monochorionic pregnancy, monochorionic twins, neonatal stroke, newborn, pachygyria, selective fetal growth restriction

## Abstract

Monochorionic twin pregnancies carry a risk of perinatal complications due to shared placental anastomoses, which can cause uneven blood distribution and lead to conditions like selective fetal growth restriction (sFGR). This case describes a monochorionic pregnancy complicated by preeclampsia and late-onset sFGR of twin B. Labor was prematurely induced and a 45% weight discordance between the twins was confirmed. Twin A adapted well to extrauterine life, but a routine cerebral ultrasound on the second day revealed a periventricular venous infarction. Subsequent brain magnetic resonance imaging (MRI) confirmed deep medullary vein thrombosis and multiple small ischemic lesions secondary to hypoxia. Twin B, born with anhydramnios, experienced several perinatal complications including resuscitation at birth and acute kidney injury. By the fourth day, twin B developed inconsolable irritability, intermittent opisthotonus, and a cortical thumb. The brain MRI showed pachygyria, suggesting a cortical development malformation. sFGR can lead to severe cerebral injuries and adverse neurodevelopmental outcomes, often impacting the larger twin due to acute in-utero blood volume shifts between the twins through placental anastomoses, while also causing brain growth restriction in the smaller twin. Balancing the risks of prematurity against the potentially serious outcomes in twins poses a significant challenge in the management of sFGR cases.

## Introduction

In recent decades, the incidence of multiple pregnancies has risen, leading to increased rates of perinatal mortality and morbidity. Chorionicity is recognized as a critical prognostic factor, with monochorionic (MC) pregnancies carrying a significantly higher risk of complications compared to dichorionic ones. This heightened risk is primarily due to a shared placenta containing vascular anastomoses between the two fetal circulations. These connections can sometimes result in an imbalanced blood distribution, which may lead to severe conditions such as selective fetal growth restriction (sFGR) or twin-to-twin transfusion syndrome [1-3].

## Case presentation

We report the case of a 30-year-old primigravida expecting MC diamniotic female twins (vertex/breech presentation), who was closely monitored at a tertiary care maternal-fetal medicine center. At 32 weeks and 6 days of gestation sFGR was diagnosed, defined by a weight discordance exceeding 25% and an estimated fetal weight for twin B at the 6th percentile. Twins A and B exhibited normal amniotic fluid indices and Doppler assessments of the umbilical artery (UA) and middle cerebral artery (MCA), with sFGR classified as type I per the International Society of Ultrasound in Obstetrics and Gynecology guidelines. However, the pregnancy was further complicated by preeclampsia without severity features at 34 weeks of gestation. At the follow-up ultrasound examination conducted at 34 weeks and 6 days of gestation, twin B exhibited severe oligohydramnios, with an UA pulsatility index above the 95th percentile and a MCA pulsatility index at the 5th percentile (peak systolic velocity of 52 cm/s in the MCA). As a result, the decision to induce labor was made, resulting in a vaginal delivery at 35 weeks of gestation. Cardiotocography revealed no evidence of perinatal fetal distress. The anatomopathological examination of the placenta confirmed monochorionicity, with the umbilical cord insertion of twin A being central and that of twin B being marginal. The birth weights of twins A and B were 2310 g and 1280 g, respectively, indicating a weight discordance of 45%. Twin A presented with a length and head circumference within the 15th-50th percentile, while twin B was below the 3rd percentile for both measurements.

**Figure 1:**
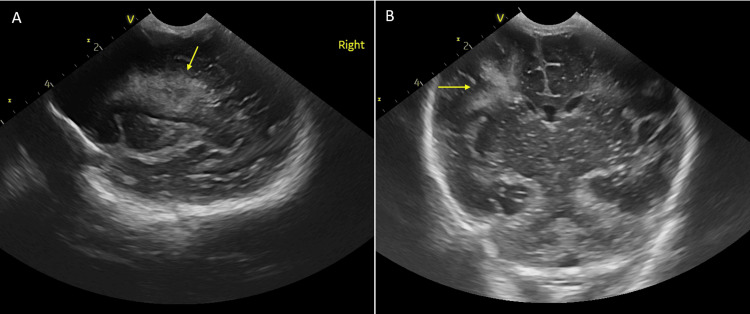
Cranial ultrasound imaging of twin A, performed via the anterior fontanelle on the second day of life A: Parasagittal view demonstrating periventricular hyperechogenicity localized to the right frontoparietal region (arrow). B: Coronal view showing a hyperechoic lesion with a radial distribution within the periventricular white matter of the right frontoparietal area (arrow), indicative of venous infarction.

Twin A adapted well to extrauterine life, achieving Apgar scores of nine in the first and fifth minutes. However, she developed a mild respiratory distress syndrome requiring non-invasive ventilation (NIV) within the first 12 hours of life, with a maximum FiO2 of 24% during the first hour. A routine cranial ultrasound (CUS) was performed on the second day of life, revealing a hyperechoic lesion spreading radially in the periventricular white matter area of the right frontoparietal lobe, consistent with venous infarction, though no intraventricular hemorrhage was present (Figure 1). The Doppler study demonstrated a resistive index of 0.76 in the anterior cerebral artery.

**Figure 2:**
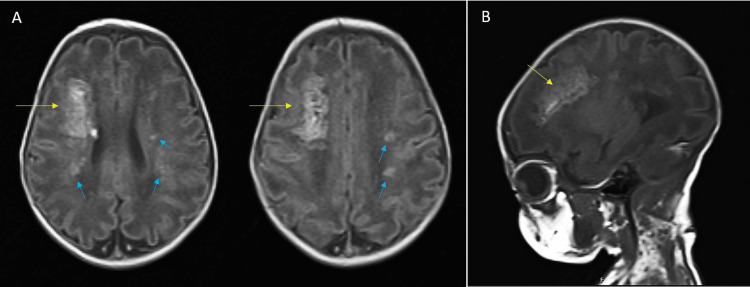
Brain MRI of twin A performed on the sixth day of life The axial T2-weighted view (A) and parasagittal T2-weighted view (B). The scans reveal thrombosis of the deep medullary veins in the right frontoparietal lobe (yellow arrows), along with additional small regions of ischemia (blue arrows), involving both internal capsules

A subsequent brain magnetic resonance imaging (MRI) scan performed on day six confirmed the diagnosis of deep medullary veins thrombosis (DMVT) on the right frontoparietal lobe, along with multiple small ischemic lesions distributed across both hemispheres, including the internal capsules, likely due to hypoxia (Figure 2). No clinical seizures were observed and the electroencephalogram (EEG) revealed no abnormalities, obviating the need for antiepileptic therapy. Tests for inherited prothrombotic disorders, including the G20210A mutation in the prothrombin gene and factor V Leiden, were normal.

**Figure 3:**
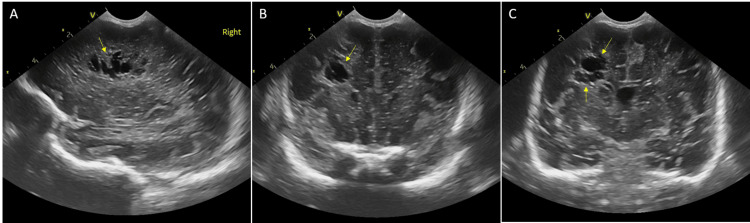
Cranial ultrasound imaging of twin A performed via the anterior fontanelle on the 21st day of life, demonstrating development of porencephalic cysts A: Parasagittal view displaying cystic formations in the frontoparietal region (arrow); B: Coronal view revealing a cyst in the right frontal region measuring 8.1 x 7.1 mm (arrow); C: Coronal view revealing cysts in the right frontoparietal region (arrows).

Serial CUS showed progression to early-stage porencephalic cysts starting from the second week, with the largest measuring 8.1 x 7.1 mm (Figure 3). Neurological examination remained normal throughout the neonatal period.

Twin B was delivered *en caul* in the context of anhydramnios, following a nine-minute interval after the delivery of twin A. The newborn exhibited hypotonia, irregular breathing, and heart rate below 60 per minute. Apgar scores were six and nine at first and fifth minutes, respectively, following successful resuscitation. Umbilical cord blood gas analysis (venous) revealed an initial pH of 7.16 (reference range: 7.35 to 7.45), base excess of -11.6 mmol/L (reference range: -2 to +2 mmol/L) and lactate level of 8 mmol/L (reference value <2.2 mmol/L), which normalized within 12 hours postpartum. She developed transient tachypnea of the newborn, requiring NIV until the third day of life with a maximum FiO2 of 23%. Additionally, she exhibited oliguric acute kidney injury (AKI) with edema and dilutional hyponatremia (minimum sodium of 123 mmol/L; reference range: 135 to 145 mmol/L), which resolved after fluid restriction and furosemide administration. Peak serum creatinine level reached 2 mg/dL on the second day, but normalized by day seven (reference range in preterm newborns: 1.0 to 1.4 mg/dL during the first two days of life and subsequent gradual decrease to 0.35 to 0.40 mg/dL). Renal ultrasound and Doppler study were both normal. Laboratory evaluation revealed an intertwin hemoglobin discordance of 7.3 g/dL, accompanied by thrombocytopenia (90,000/μL; reference range: 150,000 to 450,000/uL) and significant erythroblastosis (635 erythroblasts per 100 leukocytes; reference value: <10 per 100 leukocytes). She also experienced intermittent hypoglycemia during the first three days of life (minimum serum glucose level of 43 mg/dL; hypoglycemia defined as serum glucose level <40 mg/dL in the first 24 hours of life, and <50 mg/dL after that period), managed with enteral feeding and adjustments in intravenous glucose administration. Parenteral nutrition was maintained until the sixth day of life, in conjunction with enteral feeding.

**Figure 4:**
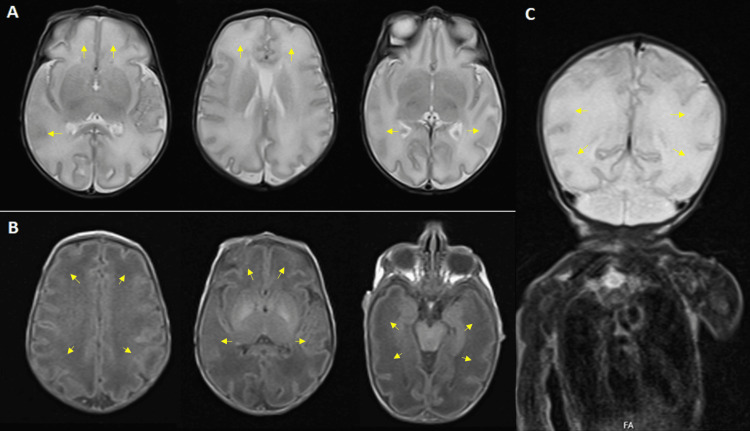
Brain MRI of twin B performed on the sixth day of life The scan revealed a simplified gyral pattern, primarily involving the frontal and temporal lobes (arrows), suggestive of a cortical developmental malformation. A: Axial T1-weighted view; B: Axial T2-weighted view; C: Coronal T1-weighted view.

CUS on the second day of life was normal. However, by the fourth day, twin B developed inconsolable irritability, intermittent opisthotonus and a persistent cortical thumb. Septic screening, including white blood cell count, C-reactive protein and procalcitonin, was negative. Blood electrolytes and glucose levels were normal and reevaluation by CUS showed no abnormalities. For the assessment of potential hypoxic-ischemic lesions, a brain MRI was conducted on the sixth day. The scan exhibited a simplified gyral pattern with frontal and temporal predominance, suggestive of a cortical development malformation such as pachygyria (Figure 4).

Maternal exposure to teratogens and prenatal infections, such as cytomegalovirus, toxoplasmosis or rubella, were excluded. EEG showed no electroclinical or electrographic seizures. The episodes of irritability resolved by day seven and no other neurological deficits were observed during the neonatal period.

Twin A was discharged after 21 days and twin B after 34 days of hospitalization. They exhibited a favorable clinical status, a normal neurological examination, and the capacity for independent feeding, with follow-up care coordinated by a multidisciplinary team comprising a neonatologist, neurologist, ophthalmologist, and otolaryngologist. Family counseling encompassed education on the potential short- and long-term neurological, developmental, and motor outcomes. These included risks of conditions such as cerebral palsy, epilepsy, and learning disabilities as well as instructions for parents on recognizing urgent symptoms such as seizures, altered levels of consciousness, or feeding difficulties, and guidance on when to seek immediate medical intervention. Upon reevaluation at three months of corrected age, the twins demonstrated normal findings on neurological examination and neurodevelopmental assessment.

## Discussion

sFGR complicates 10-15% of MC pregnancies [3]. The growth-restricted fetus typically has a smaller placental share and often exhibits a velamentous cord insertion, reducing blood supply and hindering its ability to reach its genetic potential [1,4]. However, compared to fetal growth restriction (FGR) in dichorionic or singleton pregnancies, the clinical outcome in sFGR is often less predictable, due to the presence of vascular anastomoses in the placenta that allow bidirectional blood flow and influence the progression of the condition [1,4].

A systematic review revealed that the incidence of severe cerebral injury in MC twins with sFGR can be as high as 33%, predominantly affecting cases with lower gestational age at birth, single intrauterine fetal death (sIUFD), or abnormal Doppler findings [5]. Notably, the larger twin was found to have nearly twice the risk of cerebral injury compared to the smaller twin, which can be attributed to two factors: sudden in-utero blood volume shifts between the twins through placental anastomoses, leading to hypoxic injury; or complications related to iatrogenic prematurity [5-7]. The detection of ischemic cerebral lesions within the second day of life, as observed in twin A, points to a perinatal etiology. DMVT, a rare cause of neonatal stroke, is more prevalent in twin pregnancies [8]. Most patients present with seizures (48%) or apnea (36%) during the first week of life, suggesting an insult occurring at the time of delivery. However, this association was not observed in our case, as aside from preeclampsia, no other perinatal risk factors for DVMT were identified, including signs of fetal distress, need for respiratory resuscitation at birth, inotropic support, hypoglycemia, or infection [8]. While a direct correlation between DMVT and sFGR has not been established, the latter is known to cause hemodynamic changes that may lead to thrombotic events. DMVT often progresses to necrosis and periventricular cysts, and depending on the lesion extension, long-term outcomes may include intellectual disabilities, behavioral problems, motor disorders, cerebral palsy, visual and hearing impairments, and epilepsy [8].

Placental insufficiency is the most common cause of late-onset FGR, leading to chronic fetal hypoxemia and reduced nutrient availability for the growth-restricted twin. This condition causes a range of perinatal complications such as AKI, hypoglycemia, thrombocytopenia, and erythroblastosis, all of which were observed in twin B. In late-onset sFGR, the Doppler study of the UA is typically normal for the smaller twin, reflecting mild placental dysfunction. However, advancing fetal deterioration can be evidenced by marked discordant growth and altered umbilical/cerebral ratios [9,10], signaling a redistribution of fetal cardiac output as a protective mechanism for brain growth. Despite this brain-sparing effect, normal brain development is not guaranteed, as deficits in brain structure and function are frequently observed in the FGR offspring [6,9,11]. Numerous studies report that growth-restricted infants have smaller head circumferences, a strong predictor of adverse neurodevelopmental outcomes, along with reduced volumes of grey matter, hippocampus and cerebellum, a decreased number of connectivity cells, delayed myelination, and a thinning cortex with altered gyrification [6,9,12,13]. Cerebral perfusion that often shifts toward the basal ganglia, at the expense of the frontal lobe, has also been described in FGR fetuses experiencing chronic hypoxia [9,11,12]. These factors potentially explain the imaging findings observed in twin B. The absence of similar findings in her MC twin also suggests that a genetic etiology is unlikely. Such structural changes are associated with long-term cognitive, motor and behavioral deficits, including lower intelligence quotient (IQ) scores, cerebral palsy, visuomotor dysfunction, attention deficits, and memory problems [6,9].

The management options for sFGR include expectant management, elective preterm birth, fetoscopic laser coagulation of placental anastomoses, or selective feticide. However, there is no international consensus on the optimal approach, and parents should be actively involved in the decision-making process to ensure a patient-centered management strategy that is respectful of cultural backgrounds. The key challenge is balancing the risks of prematurity-related complications with the potential for severe outcomes, such as sIUFD or cerebral injury in both twins [1,2,4,9]. While sFGR is primarily associated with restricted brain growth in the smaller twin, it can also result in severe cerebral injury in the larger twin, ultimately affecting neurodevelopmental outcomes for both.

## Conclusions

Despite vigilant monitoring and timely intervention, the twins in this case study experienced severe neonatal complications, including multiple ischemic cerebral lesions in the larger twin and brain growth restriction in the smaller one. Neurodevelopmental outcomes are possibly further impaired due to the effects of prematurity. This report underscores the complexity of managing sFGR in MC pregnancies, where placental insufficiency and vascular anastomoses contribute to unpredictable antenatal consequences. Additional research is necessary to refine management strategies and improve prognoses for MC twins affected by sFGR. 
